# Imitation Learning-Based Performance-Power Trade-Off Uncore Frequency Scaling Policy for Multicore System

**DOI:** 10.3390/s23031449

**Published:** 2023-01-28

**Authors:** Baonan Xiao, Jianfeng Yang, Xianxian Qi

**Affiliations:** School of Electronic Information, Wuhan University, Wuhan 430072, China

**Keywords:** imitation learning, uncore frequency scaling, performance-power trade-off, multicore processor, machine learning

## Abstract

As the importance of uncore components, such as shared cache slices and memory controllers, increases in processor architecture, the percentage of uncore power consumption in the overall power consumption of multicore processors rises significantly. To maximize the power efficiency of a multicore processor system, we investigate the uncore frequency scaling (UFS) policy and propose a novel imitation learning-based uncore frequency control policy. This policy performs online learning based on the DAgger algorithm and converts the annotation cost of online aggregation data into fine-tuning of the expert model. This design optimizes the online learning efficiency and improves the generality of the UFS policy on unseen loads. On the other hand, we shift our policy optimization target to Performance Per Watt (PPW), i.e., the power efficiency of the processor, to avoid saving a percentage of power while losing a larger percentage of performance. The experimental results show that our proposed policy outperforms the current advanced UFS policy in the benchmark test sequence of SPEC CPU2017. Our policy has a maximum improvement of about 10% relative to the performance-first policies. In the unseen processor load, the tuning decision made by our policy after collecting 50 aggregation data can maintain the processor stably near the optimal power efficiency state.

## 1. Introduction

The rapid development of modern communication technology brings faster data transmission speed and smaller transmission delay and also accelerates the construction of the communication network of everything connected. The increasing number of devices accessing the Internet brings huge data traffic, which leads to higher and higher computing power requirements for servers in data centers. The number of processor cores is gradually increasing with the performance requirements, and multicore processors have become a major architectural component of server computing systems. Multicore processors allow multi-threaded load work to run simultaneously on their available compute cores, thereby increasing the overall computational performance of the processor. However, high performance for servers means massive levels of power consumption. While processor architectures are moving toward highly parallel processing, the server industry as a whole is facing the problem of huge power consumption.

As the processor design architecture is updated, processors are divided into core and uncore. Simply put, uncore refers to components other than the compute core and the core’s private cache slice, including memory controllers, shared cache slice, and other important components. It has a vital impact on core computing, cache read/write, and memory access actions. Using the McPAT modeling framework on Niagara processors to evaluate the power consumption of core and uncore components, the researchers show that in a 16-core system with 32 MB of shared cache, the power consumption of the memory controller and shared cache accounts for 27% of the total power consumption of the chip [1]. As components such as shared cache and memory controllers increase in importance in processor architectures, uncore power consumption as a percentage of overall processor power consumption is increasing noticeably [2,3,4]. All of these studies show that the issue of uncore power consumption should not be underestimated.

Modern processors internally decouple the core and uncore frequency domains and provide a separate frequency domain for each core, allowing them to support single-core Dynamic Voltage Frequency Scaling (DVFS). There are two main trends in DVFS policy research for different architectures with different types of cores. One trend is to evolve from feedback-based single-step policies to machine learning (ML) policies, while the other is to evolve from offline to online learning policies. Feedback-based policies are limited by the magnitude of configuration changes and can only be adjusted in the adjacent configuration state space. In contrast, the offline ML policy completes modeling based on a sufficient amount of data with a known processor load and maps the processor state to the optimal core configuration at runtime. However, this policy of modeling based on known knowledge does not apply to unseen processor loads, so the research on dynamic power management policies for processors based on online learning has become the current focus of the industry. Imitation learning (IL) [5,6] has gradually become a research hotspot for dynamic power management policies thanks to its stronger supervised signal, higher sample utilization, and superior learning efficiency compared to reinforcement learning (RL) [7].

The uncore also has a separate adjustable frequency domain, which means that the processor’s power efficiency can be improved to a greater extent by controlling the uncore frequency. Current research on uncore frequency scaling (UFS) policies has been designed for different objectives, mostly focusing on offline learning. Some researchers emphasize the extensibility of feedback-based policies under unseen load and record the results of real-time operation of the policies as online training sets to feed artificial neural network (ANN) models for online learning [8]. Such a policy, however, will take a lot of time in data collection, and combined with the aforementioned step-limiting problem of the feedback policy, the ANN model has limitations for learning the entire input space distribution.

**Figure 1:**
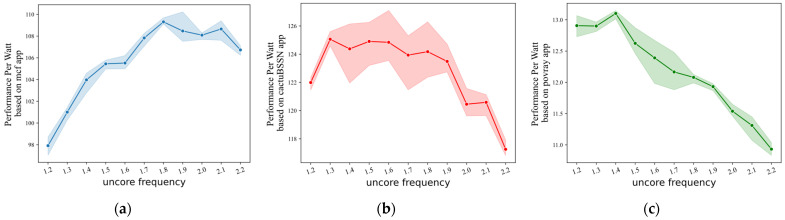
Impact of uncore frequency on processor PPW for different benchmark apps: (**a**) mcf; (**b**) cactuBSSN; (**c**) povray. The shading in each subplot indicates the fluctuating range of PPW values at different uncore frequencies.

Macroscopically, UFS policies for multicore systems have an overall goal, such as power-aware and maximum power savings with a performance penalty. To maintain performance while respecting power overhead, designers of communication and computing systems have begun to focus on power efficiency [9,10]. Thus, the latter becomes the main goal of the current UFS policy research, and the degradation index of processor performance can be left to the user to decide. With this goal in mind, the UFS policy continuously reduces the frequency within performance constraints to ensure minimal power consumption. There is a potential problem with such a tuning decision. In most cases, lowering the uncore frequency means that processor performance and power consumption will both drop. However, the drop in both is not stable, meaning that saving a certain percentage of power consumption may result in losing a larger percentage of performance. To verify this, we conduct experiments under three different load scenarios, pulling up the uncore frequency from 1.2 GHz to 2.2 GHz with the core frequency fixed, and recording the results of the processor’s response to the load. We use Performance Per Watt (PPW) to represent processor power efficiency, which is the ratio of performance to power consumption. From Figure 1, we can see that the power efficiency of the processor does not show a significant negative correlation with the uncore frequency. Under mcf load, the higher power efficiency of the processor corresponds to an uncore frequency range of 1.8–2.1 GHz, and a continuous reduction in uncore frequency will result in a continuous decrease in power efficiency. If the processor subsequently encounters a high demand load, the device latency caused by activating the electronics in the low-frequency state in response to the load may have a more significant impact.

To tackle the above problem, we propose a novel imitation learning-based UFS policy (called UFS_IL) for multicore systems, which aims to optimize the processor’s power efficiency to the maximum extent at runtime. Specifically, we first construct an offline UFS learner policy. Based on the previously known load applications in the experimental phase, we construct the learner training dataset with the relevant processor events from different underlying register statistics as the input metadata and match the optimal uncore frequency for each input state as the label for supervised learning. Meanwhile, we use the input metadata and uncore frequency as joint inputs to build a predictive model of processor performance and power consumption. The two are combined as an expert model to provide schematic data for learner policy in the online learning phase. Importantly, the learner model and the expert model are fine-tuned according to the DAgger [11] algorithm, and the design saves the learning cost of requesting expert online annotation in imitation learning. The learner policy continuously mimics the schematic data to enhance generality under unseen processor loads. Our major contributions are as follows.

1. We collect offline training datasets and obtain one UFS policy with 99% classification accuracy and three models with 99% prediction accuracy. The latter three constitute the expert model and optimize the learning efficiency by converting the learning cost of requesting experts for new data annotation into fine-tuning of the prediction model during the online learning phase;
2. We implement uncore governors based on the idea of dynamic power management governors already in use and compare them with the UFS_IL policy under the same load conditions. It is found that the processor performs best in terms of power efficiency under the latter control.
3. Online imitation learning improves the generality of the UFS policy for unseen loads. Experiments show that after collecting about 50 aggregation data, the tuning selection of the UFS_IL policy maintains the processor’s power efficiency under unseen load at near-optimal levels.
4. The remainder of this paper is as follows. Section 2 discusses the related work. Section 3 elaborates on the problem. Section 4 introduces the UFS_IL policy. Section 5 describes the experimental procedure. Finally, the conclusion is given in Section 6.

## 2. Related Work

The frequency scaling policies for processor uncore are usually investigated in two phases, the first phase models the correlation between uncore versus processor performance and power consumption, respectively. The literature [12] analyzes the memory bandwidth, last-level cache (LLC) missing rate, processor power consumption, and test completion time when the processor is under three main load types. It is concluded that reducing the uncore frequency does not have a significant impact on the processor performance when the memory power consumption remains stable. Therefore, the change in memory power consumption can be used directly to characterize the impact of uncore frequency changes on processor performance. The literature [13] identifies that the problem of processor performance degradation under cache type load is related to the size of the core’s private cache blocks. When it is large enough, the core has less craving for the shared cache. Literature [14] finds that high cache reuse rates may also lead to low LLC miss rates, so the authors combine the number of computational instructions loaded by the core to more accurately characterize the impact that uncore frequency changes have on processor performance. The literature [1] uses shared cache slices as the unit of research and uses the ratio of cache slice activations to evaluate the potential for overall cache power degradation. It is also combined with the number of instructions loaded per cache slice to fix the impact of low cache reutilization. The literature [8] measures the access latency and the number of load instructions of LLC and combines it with the core private cache miss rate to complete the modeling. As mentioned above in the literature, the uncore contains many components, each of which affects the performance of the core to a greater or lesser extent and with varying weights. The most influential components are the last level cache and the memory controller, so we combine the events related to both of them as well as the events related to the core to comprehensively characterize the processor state.

The next phase is focused on building the UFS policy. The literature [12,15,16,17] uses a feedback-based single-step tuning-type algorithm for the design, where [12,15,17] decides the next action based on the impact of the current time-step uncore tuning action on the processor performance and power consumption. The literature [16] similarly evaluates the actual impact brought by the current action and decides on the subsequent action based on the feedback state. This single-step feedback algorithm is simple and easy to understand, but the adjustment action is incremental. Limitations in search range and speed cause this policy to be less than optimal for load scenarios that require large frequency changes to respond. Further, the UFS policy is described as a mainstream problem in the field of ML, the regression problem [8,13,14], which is a statistical analysis problem that studies the relationship between a set of random variables and another set of random variables. The regression model-based solution accepts multidimensional inputs, typically from the server’s underlying register statistics of relevant events. It then evaluates the optimal performance and power consumption of the current state in the entire uncore frequency domain, and pairs the input state with the optimal uncore frequency as a piece of training data. This solution strongly relies on the sample complexity, i.e., the range of distribution of the training dataset. When the load orientation is fixed, the model can map different states to the optimal frequency configuration very accurately as long as the distribution of the collected data is wide enough. However, the runtime load is highly variable, which makes it difficult to cover the data complexity required to train the model, so the UFS policy based on ML regression models is suitable for known application scenarios.

In the research on DVFS policies for processor cores, solutions for online learning are gradually gaining importance to allow servers to determine the optimal processor configuration based on unpredictable real-time loads [8,18,19,20,21,22,23,24,25,26,27]. As noted in [8] for online scenarios, the model uses a PI controller to make decisions and collect data for training the ANN model. PI controller is also a feedback-based single-step regulation controller, and the sample distribution of the training data collected based on it also suffers from a lack of wide range. RL has also been used recently to solve the problem of finding the optimal processor configuration dynamically [18,19,20,21,22]. However, when the output dimension increases, the dimensionality of the entire problem space rises exponentially. The model needs to try different actions at each state to make a better decision, which leads to the fact that the model will require a large amount of data and time for training. Further, online imitation learning-based methods for processor power control have been proposed [23,24,25,26,27]. This approach constructs a learner policy trained in a supervised learning style based on expert demonstration data with the goal of mimicking the expert’s behavior in similar scenarios. The literature [23,25] trains the expert model on all known applications and the learner model on some of them. The ability of the expert model to guide is represented by the difference in the data distribution between the two, without considering the performance of the expert model under unseen applications. The literature [24] considers the update of the expert policy, but it models the expert model separately from the online performance-power model, which increases the complexity of the whole system. The literature [26] introduced online feature selection and adaptive forgetting factors to optimize the learning efficiency of the strategy against unknown loads. However, repeated feature selection in similar load groups with frequent switching is more harmful than beneficial. The literature [27] proposes an online performance modeling approach for GPUs that alleviates the cost of offline learning.

To sum up, both advanced UFS policies have certain drawbacks. The feedback-based policy is limited by the tuning action, which can only increase or decrease the current frequency. The machine learning-based offline policy performs well on training data, but it cannot achieve good generality on unseen workloads. Therefore, in this work, we adopt the imitation learning idea to design the UFS policy. Its advantages of requiring fewer data and time to learn near-optimal policies compared to RL are strongly in line with our requirements for UFS policies. We also shift the learning focus of the UFS policy to optimize power efficiency. In combination with the DAgger algorithm, we use the aggregated data to simultaneously optimize the expert model as well as the UFS learner policy to improve the generality of the UFS policy for unseen processor loads.

## 3. Problem Setup

Consider a multicore processor system with $N_{a}$ cores and the core frequency fixed at the highest value. All cores on the same socket share uncore resources starting from the Intel Haswell processor microarchitecture, so the solution space dimension of the problem decreases is unidimensional. Without loss of generality, we assume that there is a total of *K* bands of uncore frequencies that can be tuned at runtime. We also assume that there are $N_{a}$ different types of benchmark applications $A=\left\{A_{0},A_{1},\ldots ,A_{N_{a}-1}\right\}$, where the applications are divided into two application clusters. One application cluster *A_train* is used to build offline policies, and the other application cluster *A_test* is used to test the generality of different policies under unseen loads.

Each control epoch runs a certain target application $A_{i}\in A$. The desired policy *π* will map the system’s real-time state $s_{t_{i}}$ to a candidate uncore control frequency $f_{j}$ for the next epoch $t_{i+1}$ (*T* control epochs in total), i.e.,

(1) $$\pi ∶s_{t_{i}}\to f_{t_{i+1}}^{j},\;\;j\in \left\{0,1,\ldots ,K-1\right\}$$

We then define the power consumption $power\left(t_{i+1},\pi \left(s_{t_{i}}\right)\right)$ and performance $performace\left(t_{i+1},\pi \left(s_{t_{i}}\right)\right)$ of the processor after implementing the frequency scaling action $\pi \left(s_{t_{i}}\right)$ given by policy π in the next epoch. Our optimization goal is to design a UFS policy that allows the processor to maintain optimal power efficiency for every epoch under its control, as shown in Equation (2). The power efficiency is calculated in a similar way as in the literature [23].

(2) π*=argmaxπperformace(ti+1,π(sti))power(ti+1,π(sti))

To maintain the processor in a highly power-efficient state under dynamic and rapidly changing load environments, any power management policy must have the ability to be analyzed quickly and implement quickly. The fast analysis depends on the frequency of data sampling, which can be accomplished by compressing the amounts of tasks within each control cycle. The latter, in turn, depends on the location of the frequency controller. The possibility of deploying the power control model to the BIOS level has been explored in our previous research [22], and the additional overhead of the data acquisition process and the communication process frequency scaling signaling to the hardware registers is very small compared to the entire power savings of the UFS control system.

## 4. Imitation Learning-Based UFS Policy

### 4.1. Imitation Learning

Our problem is to dynamically select the uncore frequency that will optimize the power efficiency of the processor based on its load variation. This is a sequential decision problem and can be described as a Markov decision process. Intelligence interacts and changes with the environment, gets feedback and implements the next action, and repeats the process throughout the decision period. In this process, intelligence aims to maximize the cumulative reward and minimize the cumulative overhead of the decision steps [5,6]. Exploratory learning, represented by RL, is a representative scheme to achieve this target. Learners in RL perform actions, get rewards and then use them as weakly supervised signals to grope in huge state space. Thus, a major problem with RL is the cost of learning. The larger the dimensionality of the state space, the larger the learning burden. In addition, a good quality and generic reward function are difficult to determine, the reward function for a certain type of load is not always applicable to other types of loads. It is also necessary to consider the problem of models falling into local optima, and these problems make it extremely difficult to design a generic reward function for a processor load space with rapid update iterations. Therefore, IL schemes that consider having an intelligent body imitate the behavior of a human expert is beginning to emerge. It has been shown that IL policies converge exponentially faster than RL [11].

Unlike exploratory learning, IL relies on supervised learning. One of the most direct IL methods is Behavior Cloning. In this algorithm, all human experts’ schematic data are provided to the learner in the form $\langle s_{i},a_{i}\rangle$, which are derived from the set *A* of actions made by the experts based on the state *S*. The learner needs to learn a policy network π(S;θ) by imitating these experts’ schematic data, and the output of the policy network differs as little as possible from the distribution of the schematic data. Equation (3) is the loss function for this problem.

(3) $$Loss\left(S,A;\theta \right)≜\frac{1}{2}{\left[\pi \left(S,\theta \right)-A\right]}^{2}$$

The smaller the loss function, the closer the decision of the policy network is to the behavior of the experts. In the process of training the learner policy, we can use the data $s_{k}$ and $a_{k}$ obtained by each uniform random sampling as input, and use such algorithms as gradient update to correct the parameters in the process of backpropagation, and finally get the policy network that minimizes the value of the obtained loss function, i.e.,

(4) $$\theta_{new}\leftarrow \theta_{old}-\beta \cdot \nabla_{\theta }Loss\left(s_{k},a_{k};\theta_{old}\right)$$

The parameter β denotes the learning rate and controls the update magnitude. From a complexity point of view, when there is an opportunity to learn an approximate estimate of a model, the learning resources and cost required are much less. Corresponding to the uncore frequency scaling problem, the expert corresponds to an uncore frequency control policy that provides a strongly supervised signal for the learner policy at each decision node in the sequential decision process. The policy will mimic these actions to learn the correlation between the input state and the action. For a given processor state, the number of adjustable uncore frequency bands is limited, which means that learning can be efficiently performed by a standard supervised learning process.

### 4.2. Challenges of Applying Imitation Learning to UFS Policy

IL relies on an adequate training dataset with the required data complexity to construct learner policies. However, due to inconsistent experimental conditions and learning targets, the datasets collected by different methods for UFS policies are not generalizable. And the processor states are countless, current human experience is far from sufficient to provide schematic data for each processor input state. To solve this problem, we simulate the performance of the processor after tuning the uncore frequency to each value to determine the label data in the current state. Specifically, we apply the same amount of load to the processor and collect power consumption and performance-related event statistics. To ensure that we get the optimal decision in the whole frequency space, we pull up the frequency from the minimum to the maximum at the current uncore frequency, compare the power consumption and performance changes brought by different frequencies, and finally select the frequency that brings the optimal power efficiency as the label. The whole process is repeated on the entire training application set *A_train* and in the full frequency domain to obtain a sufficient amount of data.

The second challenge is the performance limitation problem. IL methods such as Behavior Cloning reduce the learning difficulty by transforming the decision problem into a supervised learning problem. However, the control policy obtained in this way learns an upper limit of knowledge that does not exceed the knowledge provided by the expert. According to the principle of supervised learning, if we train a conditional distribution *p(a|s)* using a schematic state with a marginal distribution *q(s)*, the conditional distribution can only achieve a good generalization performance on *q(s)*. There are two mainstream solutions to the problem of poor generalization ability. One solution is to compress the states in the decision space to reduce the dimensionality of the whole space, thus reducing the complexity of the state distribution in the physical world. Another solution is DAgger, which executes the policy in an unseen environment and collects new metadata, then requests experts for annotation. After that, a new round of Behavior Cloning is performed based on the aggregated schematic dataset. DAgger makes good use of the state distribution difference between the new environment and the training data, and it can achieve a far greater generalization capability than the general Behavior Cloning algorithms. Importantly, DAgger comes with strong theoretical guarantees for learning a policy that is very close to Oracle policy $\pi^{*}$ in a small number of iterations [6]. However, continuously requesting experts for data annotation imposes a significant update burden. Intuitively, if a rule could be learned from samples and used as a supervised signal, the number of samples required and the cost of updates would be greatly reduced. Based on this idea, we use a frequency controller based on performance-power prediction models to provide schematic data as an expert. And the work of requesting data annotation from the expert is transformed into fine-tuning the prediction models, which greatly improves the efficiency of model update iteration.

### 4.3. UFS_IL Policy Framework

**Figure 2:**
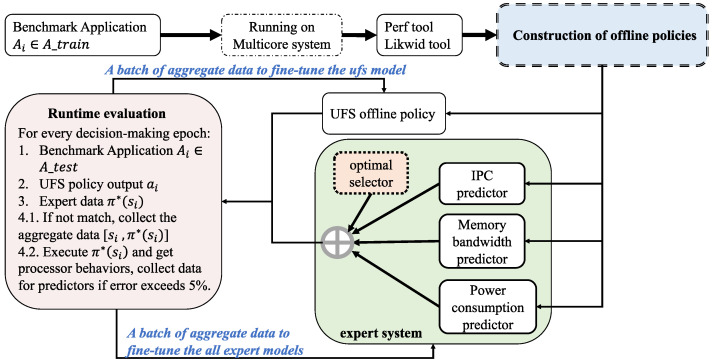
Imitation Learning-based UFS policy framework.

The process of constructing the UFS_IL policy can be divided into two phases, and the whole process is shown in Figure 2. We train the initial UFS policy and three prediction models for the *A_train* application set in the offline phase. In the online phase, we perform runtime evaluation and collect aggregation data for the *A_test* application set. The offline models are fine-tuned batch by batch to gradually increase their generality for unseen processor loads. We then describe the details for both parts.

#### 4.3.1. Offline Construction

Throughout the construction process, we run different benchmarks on the processor to simulate different types of real loads, and then run statistical tools to get the uncore-related events as input metadata. The list of selected events is shown in Table 1. Then the frequencies are switched across the uncore frequency domain and the optimal frequency value is selected as the label in supervised learning. Prior approaches use similar frameworks to construct offline UFS policies [23,24].

For the construction of the predictors, we adopt a stochastic path-based data acquisition method to optimize the whole process. After collecting the input metadata, we randomly switch the uncore frequency and add the value to the input. Then we get the processor behaviors (including IPC, memory bandwidth, and power consumption) after the frequency change and store the data as $\langle [s_{i},f_{i+1}],IPC_{i+1}\rangle$, $\langle [s_{i},f_{i+1}],mem\_bandwidth_{i+1}\rangle$ and $\langle [s_{i},f_{i+1}],power_{i+1}\rangle$, respectively. Such a data acquisition method simulates the operation of a real frequency tuning policy and respects the independence of different processor behaviors. After that, we use the learner model in supervised learning to learn the mapping relationship between states and labels. We construct four models for the whole offline process, namely UFS offline policy, IPC prediction model, memory bandwidth prediction model, and power consumption prediction model. The latter three are used by us to construct the expert model.

#### 4.3.2. Runtime Evaluation and Data Aggregation

The expert policy $\pi^{*}$ consists of three processor behavior prediction models and an optimal selector, and the output of it can be represented by Equation (5). Considering the important impact of uncore on the computational and memory access behavior of the processor, we combine the literature [15,23] to model the performance of the processor under this problem. The role of the optimal selector is to choose the frequency value corresponding to the highest power efficiency that the processor can achieve by changing the uncore frequency in the current state $s_{i}$.

(5) $$\pi^{*}\left(s_{i}\right)=\underset{f\in \left\{f_{0},f_{1},\ldots ,f_{K-1}\right\}}{\mathrm{argmax}}\frac{IPC\left(s_{i},f\right)*mem\_bandwidth\left(s_{i},f\right)}{power\left(s_{i},f\right)}$$

The initial UFS policy needs to analyze the unseen load in the online phase and continuously give the optimal tuning action to maintain the processor’s power efficiency at a high level. This target is achieved by the DAgger algorithm, but as mentioned before, DAgger algorithm requires constant requests to the expert for data annotation, which has an impact on the learning efficiency in the online phase. Also considering the extremely high requirement of accurate labeling of unseen data in the UFS problem, we split the expert model and insert control rules, and the problem of accurate labeling of new data is transformed into a problem of fine-tuning different models. The rules give the optimal supervised signal as long as the prediction models are guaranteed to be accurate. Besides, fine-tuning means that the model learns for the true data distribution on the basis of the current training, extending its already available learning capability to new similar tasks.

**Figure 3:**
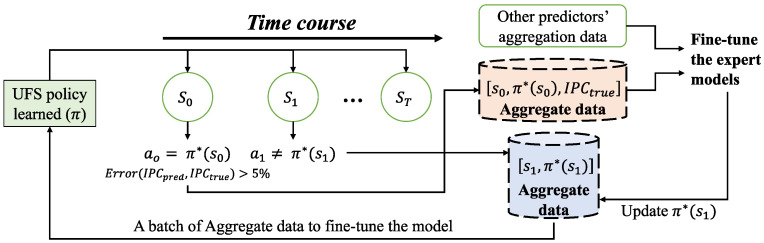
Data Aggregation Framework for Imitation Learning-based UFS policy.

The whole process of data aggregation is shown in Figure 3. Specifically, we run the application in the *A_test* application set on the processor to simulate the unseen load. The decision actions of the UFS policy and the expert policy are then compared at each decision epoch, and data is collected for the UFS policy if they do not match. We run the tools after the frequency change to get the real behavior data of the processor. If its error with the data predicted by the predictors is more than 5%, it is included as aggregation data for the expert models. Considering the limitations of the expert’s schematic output $\pi^{*}\left(s_{i}\right)$ in the initial period, we update the aggregation dataset of the UFS policy after the expert model fine-tuning is completed. And then, the policy is fine-tuned batch by batch to enhance its generality.

## 5. Experimental Evaluation

Our experiments are conducted in a multicore system with an Intel Xeon Gold 5118 CPU [28]. The system is equipped with 12 cores, each of which contains two levels of private cache and shares a third level of cache. Since core-related processor configurations are out of the scope of this research, hyper-threading and Turbo boost techniques are disabled in the experiments, and core frequencies are limited to the highest values. The uncore frequency variation range is from 1.2 GHz to 2.2 GHz, which can be tuned by setting the BIOS configuration or directly configuring the relevant MSR registers. However, due to the limited length of the registers, the frequency values that can be represented are limited, and the adjustment step is set to 100 MHz, with eleven steps.

We select nine benchmark applications from SPEC CPU2017 [29] and divide them into two application sets, *A_train* for building UFS policy and prediction models, and *A_test* for online learning to evaluate the generality of UFS policy. Table 2 describes all applications. In addition, we use the Perf [30] tool to get statistics on uncore-related events. Perf is a system performance analysis tool built into the Linux kernel source tree and works by reading the counter values in the processor’s performance monitoring unit to get statistics on related performance events. As for the performance of the processor under a specific load, we use the Likwid [31] tool to read it. This tool reads the hardware performance counters to get the statistics of some event groups and uses them as a basis to calculate a particular behavior.

### 5.1. Offline Models Evaluation

The construction process of the UFS policy is slightly different from that of the processor behavior prediction model, as the former should be classified as a classification model due to the different types of supervision labels, while the latter belongs to a regression model. Taking the power consumption prediction model as an example, we evaluate the performance of different machine learning regression models under the UFS dataset, as shown in Table 3.

We compare different regression algorithms in terms of R-Squared (R2), Mean Squared Error (MSE), Root-Sum-Squares (RSS), and training time with the same amount of data. The decision tree-based random forest regressor is found to achieve optimal prediction results with the shortest training time. Different from offline machine learning models, the model construction and training time are important for a frequency control policy that runs in real-time. It directly affects the length of the decision epoch and the process of online fine-tuning. The shorter the decision epoch, the finer-grained sequence of frequency regulation actions can be obtained at the same time, and the more flexible it can be to cope with the actual load. Therefore, we use a random forest regressor based on five decision trees to fit the relationship between the input metadata and the labels. All offline models yield R2 determination coefficients close to 1 as well as very small MSE.

### 5.2. Compared Power Efficiency with Different Advanced UFS Policies

In this section, we will compare the performance of the UFS_IL policy with two advanced UFS policies on fixed load sequences. DUF [15] and ML_guided [13] policies represent two different design ideas. They are similar in design objectives, and we briefly describe these two approaches below.

- DUF

The idea of the approach is built on a user-specified acceptable performance degradation index *θ*. Faced with different types of processor loads, DUF first ramps up the uncore frequency to the maximum and then gradually adjusts the frequency according to specified rules to save power consumption while meeting performance requirements. The rules are based on Δ*flops*, Δ*memory_bandwidth*, and Δ*llc_bandwidth*, i.e., the change in the three metrics over the two intervals before and after. This is a typical single-step feedback policy, where the decision action in the next time interval depends entirely on the outcome of the action in the current time step. It can be interpreted as a reward mechanism for immediate actions. The degree of trust in the next decision interval is determined based on the impact of the current action. If trusted, the same action is chosen, and if not, the opposite action is chosen.

In the specific experiments, we use the Likwid tool to obtain the processor characteristics metrics for each decision period and then choose to turn up, lower, or set the uncore frequency to the maximum value depending on its variation between adjacent decision periods.

- ML_guided Policy

The policy constructs two evaluation metrics, *A_Socre* and *S_Score*, both of which are represented by the statistical values of the underlying register sets on the amounts of relevant events triggered. The former indicates the thirst of each core for uncore resources, while the latter complements the impact of the size of private cache slices on the thirst of uncore resources. ML_guided policy builds two models, the first of which learns the mapping relationship between the processor state and the uncore frequencies. The obtained uncore frequencies are then united with the inputs of the previous model, corresponding to the power consumption on a case-by-case basis. A random forest learner is used to build the relational model, and the decision is completed based on the before-and-after performance change Δ*P* as well as the predicted power consumption.

In the specific experiments, we collect input features during each decision period and then read the current uncore frequency of the processor from MSR as the output label of the first model. The uncore frequencies are then incorporated into the input features and the power consumption obtained from the Likwid tool is used as the output label to train the second model.

The DUF policy focuses on lightweighting and the evaluation of the effect of immediate actions, while the ML_guided policy focuses on the generality of the known load space. And for the overall optimization goal, both are consistent, i.e.,

(6) $$\begin{array}{c} \pi^{*}=\underset{\pi }{\mathrm{argmin}}\overset{N}{\underset{0}{\sum }}power\left(t_{i}\right)\\ Subject\;to:\frac{performance\left(t_{i}\right)}{performance\_max}<1-\theta \end{array}$$

where $power\left(t_{i}\right)$ and $performance\left(t_{i}\right)$ denote the power consumption and performance of the processor after tuning during a decision period, *θ* denotes the performance degradation index, and *performance_max* denotes the maximum performance.

**Figure 4:**
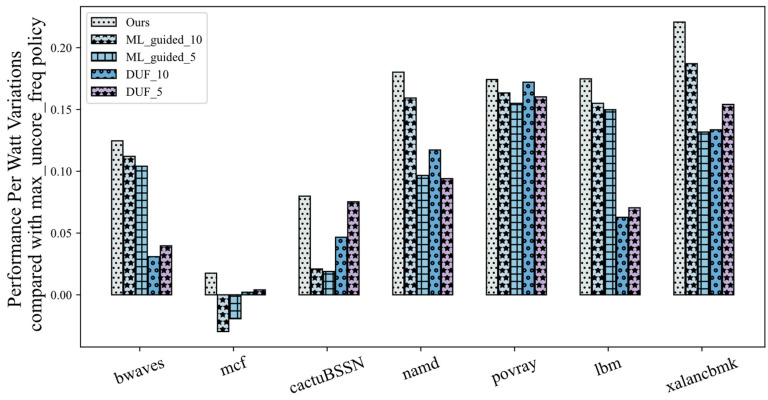
PPW variations of different models under different benchmark apps.

We evaluate the above policies on a sequence containing all applications of the *A_train* application set, repeated five times for each application. And two *θ* values of 0.1 and 0.05 are provided for the DUF and ML_guided policies with names like DUF_10 and ML_guided_5 to identify the policies with different parameters. The results are shown in Figure 4.

The results of all models are compared to the max_uncore_freq policy, which is generally the default UFS policy set by conventional processors. As we can see from the results, our UFS_IL policy achieves optimal PPW performance under all load applications, with an 8% to 22% improvement relative to the max_uncore_freq policy. There is also a certain improvement relative to the performance-first UFS policy. Under the cactuBSSN application, the PPW under the UFS_IL policy control has about a 6% improvement relative to the ML_guided_10 policy. Under the lbm application, the PPW improvement relative to the DUF_10 policy is about 10%.

At different decision nodes, the DUF policy moves step by step toward power savings based on the change in processor performance over adjacent time intervals. While the ML_guided policy can jump directly in the direction of the most power savings based on the performance prediction for the next time step, which is the main difference between the two. However, as shown in Figure 1, the PPW values for different load applications are quite different during the uncore frequency drop. This results in better performance of the DUF model in cactuBSSN-type applications and better performance of the ML_guided policy in bwaves-type applications. The expert model we designed selects the optimal PPW tuning direction based on the predictor and the optimal selector. And it can accurately predict the performance of the processor at the next time step again after the uncore frequency change to provide a continuous and effective supervisory signal. Thus the UFS_IL policy can make the optimal behavior at each decision node to bring the optimal reward.

**Figure 5:**
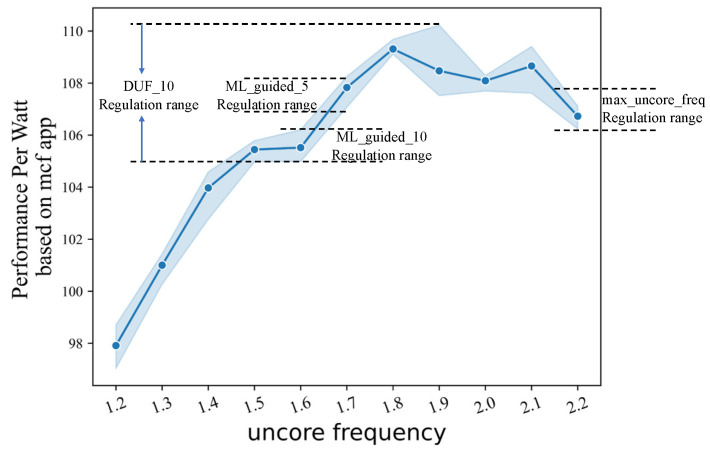
Variation of PPW at different uncore frequencies in the mcf app and different models’ regulation ranges.

Another point worth analyzing is that it seems the PPW of the processor under the control of both the ML_guided policy and the DUF policy is not significantly better than the max_uncore_freq policy under the mcf application, or even worse. Analyzing the specific situation, we find that the processor is more power efficient by keeping the uncore frequency at its maximum value. If the uncore frequency is continuously reduced within the allowed performance drop, the power efficiency of the processor increases and then decreases, as shown in Figure 5. ML_guided_10 policy maintains the uncore near the frequency allowed at the lowest point of the performance requirements, while the ML_guided_5 policy has a slightly higher power efficiency due to the higher performance requirements and the increased value of the lowest frequency allowed. Similar to the effect of the decline index *θ* on the ML_guided policy, the DUF_5 policy achieves a better power efficiency compared to the DUF_10 policy due to the reduced frequency range. The impact of the different policies on this application significantly demonstrates the problem of performance-first UFS policies, which is exactly the optimization point highlighted by the performance-power trade-off model. A certain percentage of power consumption is saved while accepting no less than that percentage of performance degradation.

### 5.3. Compared Power Efficiency with Different Power Management Governors

The actual runtime state does not require the processor to maintain the maximum frequency at all times. To save CPU power and reduce heating, the Linux kernel developers defined a framework, the CPUFreq system, which dynamically adjusts the CPU frequency according to the current CPU load state. The governor of this system selects the appropriate frequency based on the detected CPU load, and then passes this frequency value to CPUFreq_driver to complete the action of frequency regulation. However, these governors are only tuned for core frequencies and do not involve the setting of uncore frequencies. We think it is meaningful to compare our designed policy with the DVFS policies that are now maturely used, so we implement a governor for UFS following the idea of existing governors, and the introduction of each governor is shown in Table 4.

**Figure 6:**
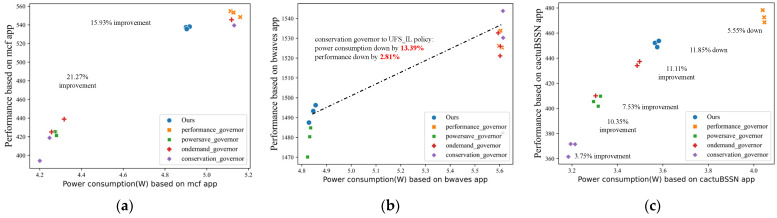
Performance and power consumption data distribution for different benchmark apps with different governors and the UFS_IL policy: (**a**) mcf app; (**b**) bwaves app; (**c**) cactuBSSN app.

We execute four different governors on the same load sequence as in the previous section to control the frequency of the processor uncore, and then compare the results with those achieved by the UFS_IL policy. It is found that the latter achieves better results under all the apps. The performance of the different models under three of the applications is shown in Figure 6.

Under mcf applications, the UFS_IL policy maintains the processor at a higher power efficiency than the better-performing power save governor, achieving a 21.27% performance improvement with 15.93% power consumption. Under the bwaves application, the processor power efficiency under UFS_IL policy control is substantially better than various governors. In this core performance-demanding application, governors choose to maintain a high frequency to ensure performance, while the UFS_IL policy chooses to reduce performance by 2.81% to reap 13.39% in power savings compared with the conservation governor. Different governors have different focuses, some for maximum power savings, some for maximum performance, and some choose to prioritize performance, but there is no governor that really focuses on the performance-power trade-off. The results for the strong core performance load of the bwaves type significantly demonstrate the advanced character of our designed UFS_IL policy regarding this point.

### 5.4. Compared Generalization on Unseen Loads

**Figure 7:**
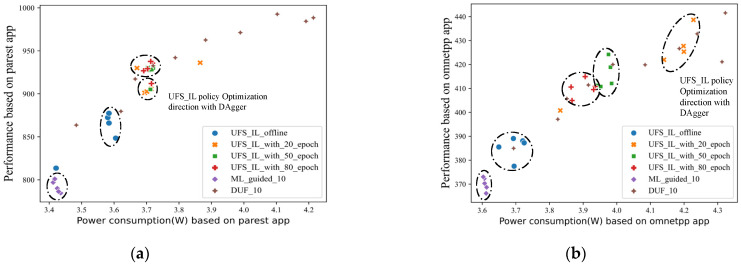
Performance and power consumption data distribution for unseen benchmark apps with different advanced UFS policies and UFS_IL policies with different amounts of aggregation data: (**a**) parest app; (**b**) omnetpp app.

In this section, we compare the performance of the UFS_IL policy with other advanced UFS policies in the unseen load application $A_{i}\in A\_test$, and the results are shown in Figure 7. This is the main improvement point of the IL-based UFS policy over other UFS policies that rely on offline training. We implement six policies, four of which are UFS_IL policies at different online learning stages, 0, 20, 50, and 80 bars respectively after fine-tuning the aggregated schematic data. The other two are the ML_guided policy and the DUF policy with a performance degradation index *θ* of 0.1. A larger *θ* implies a larger uncore frequency tuning range.

The ML_guided policy relies on the uncore frequency prediction model and power consumption prediction model. However, they are completely inaccurate for predictions of unseen load applications, since the new data distribution and the corresponding relationships are not in the knowledge base of the policy. The lowest uncore frequency in the wrong performance range is sufficient to meet the performance requirements, so the ML_guided policy tends to choose the lowest uncore frequency on unseen loads. The DUF_10 policy can be targeted according to processor behavior changes, moving toward minimal power consumption within the exact processor performance range. However, its limited pace of advancement leads to a gap in overall processor behavior relative to the UFS_IL policy.

The improvement brought by the DAgger algorithm to the UFS_IL policy is evident. After collecting 20 aggregated data, the predictors of the expert model completed the initial fine-tuning and thus the schematic data it gave are more reliable. In Figure 7 we circle the decision regions of different policies, and we can find that the decision regions of the UFS_IL_with_50_epoch policy and the UFS_IL_with_80_epoch policy are very close to each other. This shows that after collecting 50 pieces of aggregated data, the predictors have further learned the mapping relationship between unseen application metadata and processor behavior. Its prediction of processor behavior is already very close to the reality and quality of the schematic data it provides goes up. Based on that, the fine-tuning of the UFS_IL policy based on the schematic data allows it to make similar decisions within the complete data distribution space for that application, improving the power efficiency of the processor.

**Figure 8:**
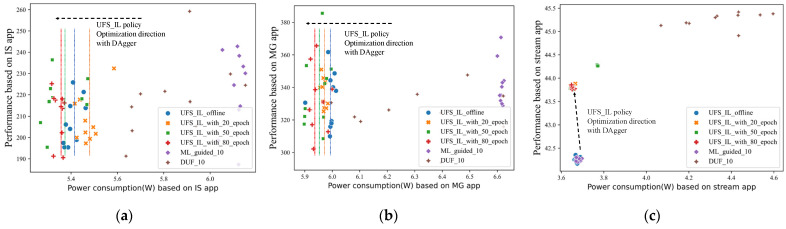
Performance and power consumption data distribution for unseen benchmark apps with different advanced UFS policies and UFS_IL policies with different amounts of aggregation data: (**a**) IS app in NPB; (**b**) MG app in NPB; (**c**) stream.

To keep the experimental results from being generalized, we also perform generalization evaluation on other unseen benchmarks. We conduct evaluation tests on IS application, MG application, and stream benchmark, the first two of which are from NAS Parallel Benchmarks (NPB). It is worth noting that the stream benchmark needs to be compiled with the *-DSTREAM_ARRAY_SIZE* parameter, which specifies the size of the test array and is the parameter that has the greatest impact on the results. We need to set this value to more than four times the processor’s highest-level cache size to ensure that the throughput performance of the CPU cache is properly tested. The results are shown in Figure 8. Also, we provide the results of the uncore frequency variation of the processor for the whole control period using the MG app as an example, as shown in Appendix A.

The test results under the stream benchmark are remarkable. As the amount of aggregation data increases, the UFS_IL policy’s tuning options get better and better, allowing the processor to maintain high power efficiency. The results under IS and MG applications are slightly scattered, due to a range of fluctuations in processor power efficiency for the same uncore configuration. We observe that the results show the average performance variation of different policies throughout the control period is not very large and the fluctuation range of power consumption is small. With this in mind, we label the average power consumption lines of different policies in the subplots. The results show that the UFS_IL policy also scales well under the NPB benchmark apps based on the DAgger algorithm. One thing to note is that the *UFS_IL_offline* policy gives better results than the *UFS_IL_with_20_epoch* policy under IS application. This is because both are not quite sufficient for fine-tuning the prediction models, while the *UFS_IL_offline* policy unexpectedly falls into a better uncore frequency range and thus achieves better results.

There is one more construction parameter worth discussing. The aforementioned power management governor in the Linux kernel system makes decisions at periodic intervals of 10–100 ms [32], which means that fifty aggregation data take 500–5000 ms to collect. That is, after a maximum of 5 s of running a repeatable unseen load clip, the expert model can complete fine-tuning and provide reliable schematic data for online learning of the learner policies.

### 5.5. Complexity Analysis

We conclude the complexity of our proposed method in this section and compare it with current advanced UFS policies. The complexity mainly refers to the construction time overhead as well as the storage overhead of the whole policy in the experimental platform. Importantly, construction time and storage space are paramount in a small system that makes quick decisions in real-time.

The DUF policy has minimal time overhead throughout the experiments due to its design advantages. This feedback-based policy does not require training in advance or data storage in the online phase. Its only overhead is the need to compute some statistical type of features and to store the feature data from the previous decision period. Overall, this feedback UFS policy has the lowest complexity, although its effectiveness is limited.

In terms of storage footprint, there is little difference between the UFS_IL and ML_guided policies. This is because both machine learning-based UFS policies require offline training to capture the mapping relationship between input features and optimal tuning actions. In our experiments, the storage size of a single processor behavior prediction model is 474–544 KB. With the inclusion of the DAgger algorithm, our system needs to store the aggregation data in the online phase. As mentioned before, the optimization goal can be achieved with 50 pieces of aggregation data, and 50 pieces of data occupy about 20 KB of storage space. On the other hand, the extra time overhead of the UFS_IL policy compared to the ML_guided policy is reflected in the online phase. the UFS_IL policy needs to feed the aggregation data to the expert model to complete the fine-tuning, which is a necessary overhead for its enhanced generality. In our experiments, the fine-tuning of the prediction model using 50 pieces of augmented data took about 22 ms.

In summary, our proposed policy achieves further optimal control of processor power efficiency with the addition of a smaller time overhead and storage overhead, ensuring a relatively proportional performance while saving power.

## 6. Conclusions

The UFS_IL policy is a novel UFS policy designed based on the idea of imitation learning, which improves the generalization of the advanced offline UFS policy to unseen processor loads. And the overall aim is set to optimize the power efficiency of the processor to avoid losing a larger percentage of performance while saving a certain percentage of power, achieving a true performance-power trade-off. Compared to the advanced single-step feedback UFS policy, the UFS_IL policy increases the regulation pace, optimizes the processor’s uncore frequency regulation speed for different loads, and achieves better overall power efficiency. Our experiments show that the processor maintains optimal power efficiency under the UFS_IL policy for known processor load applications, with an 8% to 22% improvement over the max_uncore_freq policy. Besides, this policy has a maximum improvement of about 10% relative to the offline supervised learning type policy and the single-step regulation type policy with a performance degradation index of 0.1. In the unseen load, our proposed policy with twenty aggregation data is comparable to the DUF policy that relies on immediate feedback. After collecting 50 aggregation data, the UFS_IL policy can make uncore frequency regulation decisions close to the real processor’s optimal power efficiency state.

Currently, our approach targets uncore frequencies. This set of methods is also applicable to other processor sequential decision problems, such as core frequency, cache optimization, etc. Our subsequent work will cascade uncore with other components, analyze the detailed relationships between these components, and further optimize the power efficiency of multicore systems.

## Figures and Tables

**Table 1:** All the uncore-related performance events we select as input features.

cycles	instructions	branch-misses
cpu-clock	llc_misses.mem_write	llc_misses.mem_read
unc_m_cas_count.all	l2_rqsts.miss	br_inst_retired.all_branches
mem_load_retired.l1_hit	mem_load_retired.l1_miss	mem_load_retired.l2_hit
mem_load_retired.l2_miss	mem_load_retired.l3_hit	mem_load_retired.l3_miss

**Table 2:** Benchmark applications we select from SPEC CPU2017.

	Application Name	Application Area
A_train	lbm	Fluid dynamics
	mcf	Route planning
	namd	Molecular dynamics
	povray	Ray tracing
	xalancbmk	XML to HTML conversion via XSLT
	bwaves	Explosion modeling
	cactuBSSN	Physics: relativity
A_test	parset	Biomedical imaging: optical tomography with finite elements
	omnetpp	Discrete Event simulation—computer network

**Table 3:** Evaluation metrics of different regression models with the same amount of training data.

Regressor	R2	MSE	RSS	Construction Time
Decision Tree Regressor	0.9973	1.761 × 10^−3^	3.397 × 10^−2^	350 ms
SVM Regressor	0.9468	5.373 × 10^−2^	1.128	446 ms
KNeighbors Regressor	0.9125	9.237 × 10^−3^	1.939	544 ms
Random Forest Regressor	0.9997	2.991 × 10^−4^	6.281 × 10^−3^	310 ms
Adaboost Regressor	0.9762	1.101 × 10^−2^	2.313 × 10^−1^	440 ms

**Table 4:** Different dynamic power management governors and their principles.

Governors	Principles
Performance governor	Always maintain maximum uncore frequency
Powersave governor	Maximize power savings
Ondemand governor [32]	The CPU load is calculated periodically. When the CPU load exceeds 80%, the frequency will be set to the maximum, otherwise, the frequency will be calculated proportionally according to the current load
Conservation governor	The CPU load is calculated periodically. When the CPU load is more than 80%, the default will be incremented at a 5% pace. When the CPU load is less than 20%, the default will be decremented at a 5% pace

## Data Availability

Not applicable.

## Appendix A

**Table A1:** Uncore frequency selections for different policies under the MG app throughout the control period. The unit of frequency is GHz.

Epoch	ML_guided_10	DUF_10	UFS_IL_offline	UFS_IL_with_20_epoch	UFS_IL_with_50_epoch	UFS_IL_with_80_epoch
1	2.2	2.2	2.2	2.2	2.2	2.2
2	2.2	2.2	1.4	1.5	1.5	1.3
3	2.2	2.1	1.4	1.5	1.5	1.3
4	2.2	2.0	1.4	1.5	1.2	1.4
5	2.2	2.0	1.4	1.5	1.5	1.3
6	2.2	1.9	1.4	1.5	1.5	1.3
7	2.2	1.8	1.4	1.5	1.2	1.5
8	2.2	1.7	1.2	1.5	1.5	1.5
9	2.2	1.6	1.4	1.5	1.2	1.3
10	2.2	1.5	1.4	1.5	1.2	1.3
